# Correlation between elevated serum interleukin-1β, interleukin-16 levels and psychiatric symptoms in patients with schizophrenia at different stages

**DOI:** 10.1186/s12888-023-04896-5

**Published:** 2023-06-03

**Authors:** Xialong Cheng, Yu Xie, Anzhen Wang, Cuizhen Zhu, Fanfan Yan, Wenzhi Pei, Xulai Zhang

**Affiliations:** 1 Affiliated Psychological Hospital of Anhui Medical University, Hefei, China; 2 Anhui Mental Health Center, Hefei, China; 3 Hefei Fourth People’s Hospital, Hefei, China; 4 Anhui Clinical Research Center for Mental Disorders, Hefei, China; 5 Department of Psychology, School of Educational Science, Anhui Normal University, Wuhu, China

**Keywords:** Schizophrenia, Interleukin-1β, Interleukin-16, Meso Scale Discovery, Psychiatric symptoms

## Abstract

**Background:**

There is increasing evidence that immune dysfunction plays an important role in the pathogenesis of schizophrenia. Meso Scale Discovery (MSD) is bioanalytical method, which can detect serum inflammatory factors in patients. MSD has higher sensitivities, capturing a narrower range of proteins compared to other methods typically used in similar studies. The present study was aimed to explore the correlation between the levels of serum inflammatory factors and psychiatric symptoms in patients with schizophrenia at different stages and investigate a wide panel of inflammatory factors as independent factors for the pathogenesis of schizophrenia.

**Methods:**

We recruited 116 participants, including patients with first-episode schizophrenia (FEG, *n* = 40), recurrence patients (REG, *n* = 40) with relapse-episode schizophrenia, and a control group (healthy people, HP, *n* = 36). Patients are diagnosed according to the DSM -V. The plasma levels of IFN-γ, IL-10, IL-1β, IL-2, IL-6, TNF-α, CRP, VEGF, IL-15, and IL-16 were tested by the MSD technique. Patient-related data was collected, including sociodemographic data, positive and negative symptom scale (PANSS), and brief psychiatric rating scale (BPRS) and subscale scores. The independent sample T test, χ2 test, Analysis of covariance (ANCOVA), the least significant difference method (LSD), Spearman’s correlation test, binary logistic regression analysis and ROC curve analysis were used in this study.

**Results:**

There were significant differences in serum IL-1β (*F* = 2.37, *P* = 0.014) and IL-16 (*F* = 4.40, *P* < 0.001) levels among the three groups. The level of serum IL-1β in the first-episode group was significantly higher than in the recurrence group (*F* = 0.87, *P* = 0.021) and control group (*F* = 2.03, *P* = 0.013), but there was no significant difference between the recurrence group and control group (*F* = 1.65, *P* = 0.806). The serum IL-16 levels in the first-episode group (*F* = 1.18, *P* < 0.001) and the recurrence group (*F* = 0.83, *P* < 0.001) were significantly higher than in the control group, and there was no significant difference between the first-episode group and the recurrence group (*F* = 1.65, *P* = 0.61). Serum IL-1β was negatively correlated with the general psychopathological score (GPS) of PANSS (*R*=-0.353, *P* = 0.026). In the recurrence group, serum IL-16 was positively correlated with the negative score (NEG) of the PANSS scale (R = 0.335, *P* = 0.035) and negatively correlated with the composite score (COM) (*R*=-0.329, *P* = 0.038). In the study, IL-16 levels were an independent variable of the onset of schizophrenia both in the first-episode (OR = 1.034, *P* = 0.002) and recurrence groups (OR = 1.049, *P* = 0.003). ROC curve analysis showed that the areas under IL-16(FEG) and IL-16(REG) curves were 0.883 (95%CI:0.794–0.942) and 0.887 (95%CI:0.801–0.950).

**Conclusions:**

Serum IL-1β and IL-16 levels were different between patients with schizophrenia and healthy people. Serum IL-1β levels in first-episode schizophrenia and serum IL-16 levels in relapsing schizophrenia were correlated with the parts of psychiatric symptoms. The IL-16 level may be an independent factor associating with the onset of schizophrenia.

## Background

Schizophrenia is a neurodevelopmental disorder caused by the interaction of genetic factors and environmental factors, and the pathogenesis may involve neurodevelopmental abnormalities, neurotransmitter disorders, and immune dysfunction [1]. Among them, systemic immune dysfunction plays an increasingly important role in the occurrence and development of schizophrenia. Evidence suggests that the severity of psychiatric symptoms in patients with schizophrenia is correlated with a variety of serum inflammatory factors [2–6]. Most of them showed a positive correlation between serum IL-1β and IL-6 levels and psychiatric symptoms [5, 7, 8]. One study suggests that the severity of psychiatric symptoms can be predicted by IL-1β concentration [5]. Animal models also suggest that the presence of schizophrenia-like behaviors may be associated with increased IL-1β levels [9].

On account of the heterogeneity of the examination methods and the different racial and ethnic groups involved, the results of the research lacked consistency, and most studies were used in multiple cases, such as ELISA [10] and Luminex [11]. Conventional detection ranges can only reach 10pg/ml–1,000pg/ml [11, 12]. The concentration distribution of protein to be measured in the samples generally ranges from a few tenths of pg to thousands of pg, so the linear range of an ELISA or Luminex kit cannot simultaneously detect high and low protein abundance. Meso Scale Discovery (MSD) is bioanalytical method, which can detect serum inflammatory factors in patients. Compared with the typical detection methods of serum inflammatory factors, MSD has higher sensitivities, which was up to 0.05pg/ml [13]. It provides a wide linear range from the Sub-peak level to tens of thousands of picograms of concentration, and all samples effectively fall within the optimal linear range for accurate determination, which has higher clinical application value [12, 14].

The present study was aimed to explore the correlation between the levels of serum inflammatory factors and psychiatric symptoms in patients with schizophrenia at different stages. It investigated a wide panel of inflammatory factors in patients with relapsing and first-episode schizophrenia. The inflammatory factors were measured using the MSD method that captured a narrower range of proteins compared to other methods typically used in similar studies. This study provides new approaches for the pathophysiological mechanism of schizophrenia and the optimization of treatment.

## Methods

### Research criteria

Inclusion criteria: (1) 18–65 years old; (2) the patient met the Diagnostic and Statistical Manual of Mental Disorders Edition 5 (DSM-V) criteria and was diagnosed with schizophrenia by two or more attending physicians with professional titles; (3) the patient did not take antibiotics, immunosuppressants, or other drugs recently and did not have any infections, such as respiratory tract infection or skin infection (within the last month); (4) participants who were taking or were not taking anti-psychotics met the inclusion criteria; (5) the first-episode group (FEG): The patient of the group had only one onset of schizophrenia; (6) the recurrence group (REG): The patient of the group had two or more episodes of schizophrenia; (7) all patients and healthy people are randomly selected.

Exclusion criteria: (1) combined with other mental diseases (such as mental retardation, etc.) or serious nervous system diseases; (2) history of psychoactive substance dependence; (3) women in pregnancy or lactation; (4) recent serious physical diseases, such as immune system diseases, etc. (within the last three months).

### General information

Patients with first-episode or relapsing schizophrenia who were admitted to the Fourth People’s Hospital of Hefei from May 2018 to December 2019 and met the research criteria were recruited as the subjects for this study. We recruited 116 participants, including patients with first-episode schizophrenia (FEG, *n* = 40), recurrence patients (REG, *n* = 40) with relapse-episode schizophrenia, and a control group (healthy people, HP, *n* = 36). The subjects themselves, or with the consent of their guardians, agreed to participate in the study. They were informed by the staff of the specific procedures in the study, as well as the possible risks and benefits involved, and signed a document giving their informed consent for the project. This study was approved by the Ethics Committee of the Fourth People’s Hospital of Hefei and approved by the Chinese Clinical Trial Registration Center (No. ChiCTR1800019343).

### Data collection

Social demographic data such as gender, age, marital status, and educational background of patients and healthy people were collected, and data such as height, weight, age of first onset, and total course of disease were collected from the electronic medical record system of patients in the hospital.

The MSD method was used to measure serum levels of IFN-γ, IL-10, IL-1β, IL-2, IL-6, TNF-α, CRP, VEGF, IL-15, and IL-16 in patients and healthy people on the day of admission or from 6:00–8:00am on the following day. Fasting venous blood was collected by ethylene diamine tetraacetic acid(EDTA)anticoagulant tubes and sent to the laboratory for separation. Centrifuged at 3,000 revolutions per minute (RPM) for 10 min, the separated serum was stored in a refrigerator at -80℃ and sent to Shanghai Uning Bio-technology Co., LTD for centralized detection after collection. Meso Scale Discovery (MSD) is a global leader in the development, manufacture, and commercialization of innovative assays and instruments for the measurement of molecules in biological samples. MSD’s proprietary MULTIARRAY technology enhances medical research and drug development by enabling researchers to profile many biomarkers simultaneously in a single sample without compromising assay performance. MSD’s technology has been widely adopted by researchers in pharmaceutical companies, government institutions, universities, and clinical laboratories worldwide for its high sensitivity, excellent reproducibility, and wide dynamic range.

Scale assessment patients with schizophrenia were evaluated at baseline (at admission) for psychiatric symptoms using 30 items Chinese version of Positive and Negative Symptom Scales (PANSS) [15] and 18 items Chinese version of Brief Psychiatric Rating Scales (BPRS) [16]. PANSS scale can be divided into four subscales, respectively positive scale (POS, possible score range 7–49), negative scale (NEG, possible score range 7–49), general psychopathological score (GPS, possible score range 16–112), and composite scale (COM, possible score range from − 41 to + 42). The higher the PANSS total score and its subscales, the more severe the mental symptoms. The BPRS scale included the total score and five factor scores, which are classified into five categories: anxiety and depression(ANDP), lack of energy (ANEG), thought disorder (THOT), activation(ACTV), and hostility(HOST). The total score reflects the severity of the disease—the higher the total score, the more serious the disease—while the factor score reflects the clinical characteristics of the disease.

### Observation indicators

The social demographic data were divided into the first-episode group, the recurrence group and the control group. Compare the differences in inflammatory factor levels in the serum of patients with the serum and analyze the correlation between inflammatory factor levels and psychiatric symptoms. To explore whether significant increases in inflammatory factors can be an independent factor in associating with the onset of schizophrenia.

### Statistical methods

SPSS 22.0 statistical software was used for data analysis. PASS11 software was used to calculate the sample size. The count data of baseline demographic and clinical characteristics was expressed as n (N%), the comparison between the three groups was conducted by χ2 test. Quantitative data of baseline demographic and clinical characteristics is represented as (Mean ± SD) and the independent sample T test was used for comparing the first-episode group(FEG) and the recurrence group(REG). After hypothesis testing, the data of inflammatory factors in the three groups were roughly in line with normal distribution, without special correction. Analysis of covariance (ANCOVA) was used for comparison of inflammatory factors between the three groups, and the least significant difference method (LSD) was used for comparison of inflammatory factors between the two groups. The *P* value was modified using the Bonferroni. Spearman’s correlation coefficient for ranked data was used to analyze the correlation between significantly increased inflammatory factors and psychiatric symptoms of patients. Binary logistic regression analysis and ROC curve were used to evaluate the significance of IL-16 level in schizophrenia.

## Results

Following a comparison of the baseline demographic and clinical characteristics among the three groups, in terms of gender (χ2=-0.71, *P* = 0.703), age (χ2=-3.33, *P* = 0.189), marital status (χ2=-5.47, *P* = 0.065), years of education (χ2= 4.52, *P* = 0.105), place of residence (χ2=-1.85, *P* = 0.400), body mass index (BMI) (t = 1.11, *P* = 0.334), age of first onset (t = 0.19, *P* = 0.846), total course of disease (t = 0.46, *P* = 0.649), PANSS total score (t = 1.65, *P* = 0.103), and BPRS total score (t = 1.46, *P* = 0.149), no statistically significant difference was found (*P* > 0.05) (see Table 1).

**Table 1:** Baseline demographic and clinical characteristics

Project	FEG (*n* = 40)	REG (*n* = 40)	HP (*n* = 36)	χ2/t	*P*
Gender					
Male	12(30)	13(32.5)	14(38.9)	0.71	0.703
Female	28(70)	27(67.5)	12(61.1)		
Age					
＜age of 45	34(85)	35(87.5)	35(97.2)	3.33	0.189
≥age of 45	6(15)	5(12.5)	1(2.8)		
Marital status					
Married	22(55)	14(35)	11(30.5)	5.47	0.065
Single	18(45)	26(65)	25(69.5)		
Education life					
≤ 6 years	9(22.5)	8(20)	2(5.5)	4.52	0.105
＞6 years	31(77.5)	32(80)	34(94.5)		
Domicile					
City	15(37.5)	17(42.5)	19(52.7)	1.85	0.400
Town	25(62.5)	23(57.5)	17(47.3)		
BMI(kg/m^2^)	23.13(3.25)	23.92(5.08)	22.60(2.96)	1.11	0.334
First onset of Disease (age)	28.5(12.0)	32.2(13.0)	/	0.19	0.846
Total course (years)	2.54(1.36)	2.70(1.30)	/	0.46	0.649
PANSS(points)	89.60(10.85)	83.43(21.02)	/	1.65	0.103
BPRS(points)	50.05(10.24)	46.93(8.91)	/	1.46	0.149

*BMI* Body Mass Index, *PANSS* Positive and Negative Symptom Scale, *BPRS* Brief Psychiatric Rating Scale

The differences in serum inflammatory factor levels in the first-episode group, recurrence group, and control group were analyzed. To explore the differences in serum inflammatory factor levels among the three groups, the results showed that serum IL-1β (*F* = 2.37, *P* = 0.014) and IL-16 (*F* = 4.40, *P* < 0.001) were significantly different. The level of serum IL-1β in the first-episode group was significantly higher than in the recurrence group (*F* = 0.87, *P* = 0.021) and the control group (*F* = 2.03, *P* = 0.013), but there was no significant difference between the recurrence group and the control group (*F* = 1.65, *P* = 0.806). The serum IL-16 level in the first-episode group (*F* = 1.18, *P* < 0.001) and the recurrence group (*F* = 0.83, *P* < 0.001) was significantly higher than in the control group, and there was no significant difference between the first-episode group and the recurrence group (*F* = 1.65, *P* = 0.612). Gender (*F* = 0.24, *P* = 0.994), age (*F* = 1.63, *P* = 0.101), education (*F* = 1.80, *P* = 0.065), marital status (*F* = 1.34, *P* = 0.213), Domicile (*F* = 1.39, *P* = 0.189), height (*F* = 1.56, *P* = 0.122), and weight (*F* = 1.52 2, *P* = 0.135) and BMI index (*F* = 1.55, *P* = 0.125) were analyzed as covariables, and there was no significant difference (see Table 2). The sample size was calculated and the significance level of statistical test was set as bilateral α = 0.05. Based on the sample sizes of the three groups, the statistical power estimation was 0.990.

**Table 2:** Comparison of plasma inflammatory factors among the three groups

Inflammatory cytokines	FEG	REG	HP	Adjusted FEG	Adjusted REG	Adjusted HP	*F*	*P*	FEG VS REG		REG VS HP	REG VS HP
	(*n* = 40)	(*n* = 40)	(*n* = 36)	(*n* = 40)	(*n* = 40)	(*n* = 36)						
IFNγ	10.61 ± 18.58	23.35 ± 55.11	58.75 ± 185.41	18.53 ± 18.50	18.48 ± 18.59	55.35 ± 22.14	1.015	0.436		0.601	0.056	0.159
IL1	1.28 ± 2.06	0.73 ± 0.79	0.50 ± 0.59	1.09 ± 0.28	0.61 ± 0.28	0.58 ± 0.34	1.56	0.129		0.402	0.169	0.573
IL1β	1.06 ± 2.80	0.18 ± 0.29	0.08 ± 0.48	1.14 ± 0.27	0.13 ± 0.27	0.02 ± 0.32	2.374	0.014^*^		0.021^*^	0.013^*^	0.806
IL2	0.31 ± 1.01	0.35 ± 0.27	0.43 ± 0.88	0.33 ± 0.13	0.30 ± 0.13	0.44 ± 0.16	0.346	0.966		0.818	0.534	0.69
IL6	1.00 ± 0.88	2.26 ± 4.54	1.15 ± 2.47	0.92 ± 0.53	2.16 ± 0.53	1.33 ± 0.63	0.541	0.858		0.067	0.832	0.115
TNFα	14.96 ± 53.73	6.27 ± 2.81	6.56 ± 18.62	17.06 ± 5.61	6.22 ± 5.64	4.27 ± 6.72	0.983	0.463		0.245	0.274	0.97
CRP	4.80 ± 1.70*10^6	3.92 ± 7.6*10^6	1.73 ± 3.70*10^6	3.99 ± 1.82*10^6	2.99 ± 1.83*10^6	3.84 ± 2.18*10^6	1.617	0.112		0.669	0.205	0.393
VEGF	122.58 ± 101.19	121.08 ± 99.56	89.14 ± 58.99	129.4 ± 15.12	122.86 ± 15.19	79.59 ± 18.10	1.16	0.326		0.94	0.107	0.124
IL15	2.45 ± 0.45	2.46 ± 0.68	8.14 ± 33.09	2.41 ± 3.17	2.26 ± 3.19	8.39 ± 3.80	0.563	0.841		0.999	0.182	0.182
IL16	307.72 ± 138.99	295.95 ± 94.88	175.61 ± 50.51	308.85 ± 17.46	307.38 ± 17.54	161.65 ± 20.89	4.406	< 0.001^**^		0.61	< 0.001^**^	< 0.001^**^

^*^ *P* < 0.05. ^**^ *P* < 0.001

The correlation analysis was performed between serum IL-1β, IL-16 levels and psychiatric symptoms in the first-episode and recurrence groups were evaluated. To explore the correlation between serum inflammatory factors (IL-1β and IL-16) with significant differences of the three groups and psychiatric symptoms. Serum IL-1β was negatively correlated with the GPS (*R*=-0.353, *P* = 0.026). In the recurrence group, serum IL-16 was positively correlated with the NES (R = 0.335, *P* = 0.035) and negatively correlated with the COM (R=-0.329, *P* = 0.038) (see Table 3).

**Table 3:** The correlation between plasma inflammatory factors and clinical symptoms

Scale and subscale scores	FEG (*n* = 40)				REG (*n* = 40)			
	IL-1β		IL-16		IL-1β		IL-16	
	*r*	*P*	*r*	*P*	*r*	*P*	*r*	*P*
PANSS	-0.176	0.279	0.169	0.299	0.187	0.247	0.241	0.134
POS	-0.021	0.899	0.034	0.834	0.185	0.253	-0.058	0.721
NEG	-0.160	0.324	0.165	0.309	0.176	0.278	0.335	0.035^*^
GPS	-0.353	0.026^*^	0.147	0.365	0.165	0.308	0.167	0.302
COM	0.068	0.678	0.092	0.572	-0.154	0.343	-0.329	0.038^*^
BPRS	0.137	0.399	0.065	0.692	0.147	0.367	0.264	0.100
ANDP	0.036	0.826	0.102	0.530	-0.031	0.851	0.102	0.530
ANEG	0.210	0.193	0.268	0.094	0.141	0.384	0.268	0.094
THOT	0.107	0.511	0.302	0.058	0.103	0.526	0.302	0.058
ACTV	0.057	0.728	-0.041	0.803	-0.017	0.915	-0.041	0.803
HOST	0.000	0.999	0.103	0.527	0.097	0.552	0.103	0.527

^*^ *P* < 0.05. *POS* Positive, *NEG* Negative, *GPS* General Psychopathological, *COM* Composite, *ANDP* Anxiety and Depression, *ANEG* Lack of Energy, *THOT* Thought Disorder, *ACTV* Activation, *HOST* Hostility

The binary logistic regression analysis was used to explore whether serum IL-1β and IL-16 levels could relate to the onset of schizophrenia. In the analysis, people with and without schizophrenia were dependent variables. The covariable variables are gender, age, Marital status, Domicile, BMI, serum IL-1β and IL-16 levels. Therefore, we established two binary logistic regression models (FEG and HP, REG and HP) respectively, and Hosmer-Lemeshow fitting degrees were 0.268 and 0.999, indicating that the models were well established. In the models, serum IL-16 levels were found to be an independent factor, which can associate with the onset of schizophrenia in both the first-episode group and control group (OR = 1.034, *P* = 0.002) and recurrence group and control group (OR = 1.049, *P* = 0.003) (see Table 4).

**Table 4:** Binary logistic regression analysis of influencing factors of schizophrenia

The independent variables	FEG (*n* = 40) and HP (*n* = 36)				REG (*n* = 40) and HP (*n* = 36)			
	OR	95%CI		*P*	OR	95%CI		*P*
		Z (0.025)	Z (0.975)			Z (0.025)	Z (0.975)	
Gender	0.285	0.032	2.508	0.258	0.130	0.01	1.682	0.118
Age	0.981	0.865	1.112	0.766	1.158	1.032	1.299	0.012
Marital status	0.445	0.045	4.439	0.491	0.191	0.008	4.475	0.304
Domicile	0.385	0.039	3.771	0.412	0.921	0.063	13.51	0.952
BMI	1.287	0.935	1.772	0.121	0.966	0.725	1.286	0.812
IL-1β	2.559	0.540	12.12	0.236	0.475	0.055	4.106	0.498
IL-16	1.034	1.012	1.057	0.002^*^	1.049	1.016	1.083	0.003^*^

^*^ *P* < 0.01

**Fig. 1:**
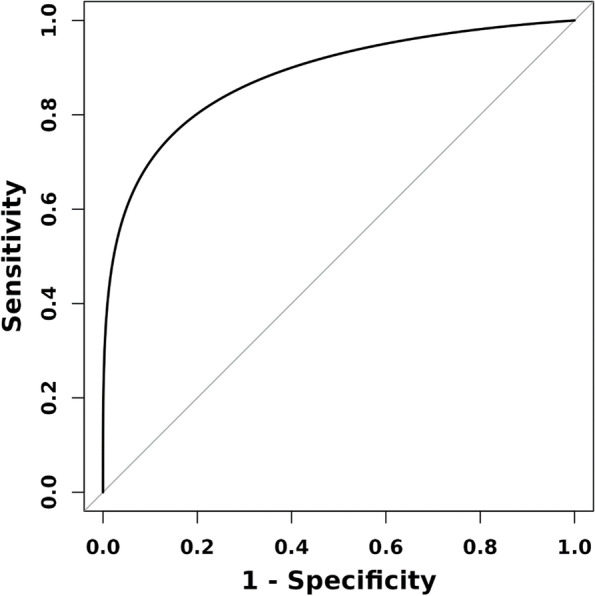
ROC curve analysis of IL-16 in FEG

**Fig. 2:**
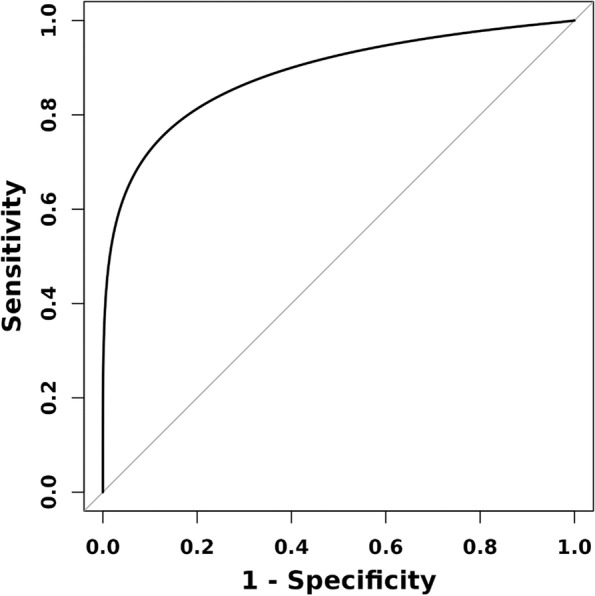
ROC curve analysis of IL-16 in REG

ROC curve were used to evaluate the significance of IL-16 level in the diagnosis and prediction of schizophrenia. ROC curve analysis results indicated that the area under IL-16(FEG) curve was 0.883 (95%CI: 0.7944–0.942), and the area under IL-16(REG) curve was 0.887 (95%CI:0.801–0.950). (see Table 5; Figs. 1 and 2).

**Table 5:** ROC curve analysis of IL-16 in schizophrenia

Index	AUC (95% CI)	Sensitivity	Specificity	Cutpoint
IL-16(FEG)	0.883 (0.794,0.942)	85.00%	80.60%	213.94
IL-16(REG)	0.887 (0.801,0.950)	75.00%	94.40%	237.81

## Discussion

At present, there is evidence that patients with schizophrenia have abnormal immune function, and the imbalance of serum inflammatory factors may be involved in the occurrence and development of schizophrenia [17, 18]. In this study, MSD was used to accurately detect the levels of serum inflammatory factors in patients with schizophrenia. Plasma IL-1β levels in patients with first-episode schizophrenia were significantly higher than those in patients with relapsing schizophrenia and healthy subjects. A number of previous studies found significantly elevated levels of IL-1β in the serum of first-episode untreated patients or adolescents [19, 20]. In addition, serum IL-1β levels were significantly elevated in both first-episode and relapsing patients [20, 21]. A meta-study found that IL-6 decreased significantly in patients with acute schizophrenia and continued to decline in subsequent treatment for acute illness, IL-6, tnf-α and CRP had often been shown to be associated with psychiatric disorders, but this positive result was not found in our results, which may also be related to inconsistencies in patient inclusion criteria, detection timing, and detection methods, etc. [22]. However, recent studies have shown that this abnormal elevated state can be gradually recovered or significantly reduced after drug treatment [23–25]. Therefore, IL-1β may be a biomarker for the onset of schizophrenia.

Research shows that IL-16 plays an important role in inflammatory diseases in the central nervous system [26]. The current study indicated that the plasma level of IL-16 in patients with first-episode and relapsing schizophrenia was significantly higher than in healthy people, suggesting that IL-16 may play an important role in the occurrence and development of schizophrenia. At present, there is a lack of research on the relationship between IL-16 and schizophrenia, but research on IL-16 and mental illness existed. One study shows that compared with the control group, the expression of IL-16 mRNA and related proteins in children with loneliness spectrum disorder significantly increased, suggesting that the expression of IL-16 may play an important role in immune changes in children with loneliness spectrum disorder [27]. A second study also found that neurocognitive symptoms in patients with depression were positively correlated with the transcription levels of pro-inflammatory factors TNF-α, IL-16, McP-1, McP-2, MIP-1-α, MIP-1 -β, TGF -β, and IFN-β [28]. These findings suggest that immune cell activation also plays an important role in neurocognitive dysfunction in depression. At present, relatively consistent studies indicate that IL-16 is significantly correlated with the pathogenesis of Alzheimer’s disease (AD) [29–32]. At the genetic level, the women who carried APOE 4 had higher levels of IL-16 in their CSF than the non-APOE carriers [33], while the men who carried APOE 4 had the opposite level of IL-8 [34]. The results showed that ChT, IL-16, IL-18, and TGF-β1 increased in ischemic cerebrovascular disease (CVD) and AD, confirming that the immune system may play an important role in the development of neurodegenerative diseases. At present, about the role of IL-16 less reported in schizophrenia, researchers found that chemokines and heavy mental illness (major depressive disorder, bipolar disorder, and schizophrenia) correlated with suicidal behavior in patients with brain dorsolateral prefrontal cortex tissue samples, and IL-16 levels increased significantly [35]. However, research on the relationship between IL-16 and schizophrenia is lacking, which needs to be paid more attention in the future.

In addition, this study also analyzed the correlation between significantly increased inflammatory factors (IL-1β, IL-16) and psychiatric symptoms. The results showed that plasma IL-1β was negatively correlated with GPS in the first-episode group. In the recurrence group, plasma IL-16 was positively correlated with NES and negatively correlated with COM. One study found that increased IL-1β and IL-6 levels are associated with positive symptoms [36]. Other studies have found that elevated IL-1β, IL-4, IL-6, and IL-8 levels are associated with the severity of psychiatric symptoms [8, 37, 38]. The association between IL-1β and symptoms was found only in patients with chronic schizophrenia but not in first-episode patients [39]. At present, IL-16 is related to the psychiatric symptoms of patients with schizophrenia, no correlation has been found between the total score of PANSS, and each factor score of IL-16 increases. On the one hand, the inconsistency of the above results may be related to the selection of research objects, the severity of the disease and the different stages of the disease. On the other hand, it also reflects the complexity of the inflammatory response of schizophrenia, the different stages of the disease, the differing severity and whether the treatment can lead to changes in the inflammatory response. In future studies, therefore, patients may need to be classified according to being treated or untreated and disease severity for a more in-depth discussion.

The current study also show that plasma IL-16 levels are an independent factor in associating with the onset of schizophrenia in both first-episode and relapsing patients. At present, many studies have explored the possibility of a variety of inflammatory factors as biomarkers that could relate to the occurrence and development of schizophrenia, but there is a lack of consistent research. There are few studies on the association between schizophrenia and IL-16. Researchers found that the interplay between dyslipidemia of high-density lipoprotein, chronic inflammation, and psychotic symptoms suggested a strong impact of inflammatory dysregulation on metabolic risk in these patients. In particular, the study clarified the potential of dysbiosis peripheral inflammatory factors as variables of clinical outcomes in patients with psychosis. The main inflammatory factors associated with clinical outcome in psychosis were IL-15, IL-13, IL-16, and IL-8, which may be associated with poor clinical outcome [40]. Consistently, several studies found that the IL-15, IL-16, and IL-8 levels in patients with schizophrenia are associated with a poor response to antipsychotic treatment, and increased IL-8 levels are significantly associated with more severe psychiatric symptoms in patients. These findings suggested that these pro-inflammatory cytokines are potential markers to identify patients with poor clinical outcome [41, 42]. The results of this study provide a new research idea for the biomarkers of the onset of schizophrenia.

The above results indicate that there is an obvious immune dysfunction in schizophrenia patients, which is generally consistent with the results of this study. However, some cytokine changes have been reported differently in several studies, and the difference in results may be related to the age, severity of disease, course of disease, sample size, types of antipsychotic drugs used and different detection reagents of the included patients. In addition, the coverage of cytokine levels and different inconsistent performances reflecting the immune abnormalities in patients with schizophrenia is very complicated, as inflammation of patients with schizophrenia and anti-inflammatory responses may be in a state of imbalance, and the imbalance of inflammation may play an important role in the process of pathophysiology of schizophrenia. In the future, it is necessary to increase the sample size and design follow-up studies to further observe the changes and mechanisms of cytokines.

The limitation of this study is that it is a cross-sectional observational study without follow-up before and after drug treatment, the influence of drugs cannot be ruled out, led to the relationship between changes of inflammatory factors and changes to psychiatric symptoms before and after treatment cannot be deeply discussed. Future studies are needed to classify patients according to their clinical characteristics, and follow-up studies should be designed to observe the changes of serum inflammatory factors before and after treatment and their relationship with psychiatric symptoms to further clarify the causal relationship and specific mechanism of inflammatory responses of schizophrenia.

## Conclusions

In conclusion, the levels of IL-1β and IL-16 are different between patients with schizophrenia and healthy subjects. Plasma IL-1β of first-episode schizophrenia and plasma IL-16 of relapsing schizophrenia were correlated with the parts of psychiatric symptoms. IL-16 levels may be an independent factor for the onset of schizophrenia.

## Data Availability

The datasets generated in this study are available from the corresponding author on reasonable request.

## References

[1] Yuan X, Kang Y, Zhuo C, Huang X-F, Song X (2019). The gut microbiota promotes the pathogenesis of schizophrenia via multiple pathways. Biochem Biophys Res Commun.

[2] Fond G, Lançon C, Korchia T, Auquier P, Boyer L (2020). The role of inflammation in the treatment of schizophrenia. Front Psychiatry.

[3] Zhang L (2019). The effect of minocycline on amelioration of cognitive deficits and pro-inflammatory cytokines levels in patients with schizophrenia. Schizophr Res.

[4] Pandurangi AK, Buckley PF (2019). Neuroinflammation and schizophrenia current topics in behavioral neurosciences Chapter 91.

[5] González-Blanco L (2019). ¿Pueden ser la interleucina-2 y la interleucina-1β biomarcadores específicos de la sintomatología negativa en la esquizofrenia?. Revista de Psiquiatría y Salud Mental.

[6] Orsolini L (2018). Protein-C reactive as biomarker predictor of schizophrenia phases of illness? A systematic review. Curr Neuropharmacol.

[7] Zhang L (2018). Minocycline adjunctive treatment to risperidone for negative symptoms in schizophrenia: association with pro-inflammatory cytokine levels. Prog Neuropsychopharmacol Biol Psychiatry.

[8] Zhu F (2018). Altered serum tumor necrosis factor and interleukin-1β in first-episode drug-naive and chronic schizophrenia. Front Neurosci.

[9] Ventura L (2020). Involvement of NLRP3 inflammasome in schizophrenia-like behaviour in young animals after maternal immune activation. Acta Neuropsychiatrica.

[10] Aydin S (2015). A short history, principles, and types of ELISA, and our laboratory experience with peptide/protein analyses using ELISA. Peptides.

[11] Sourial S, Marcusson-Ståhl M, Cederbrant K (2009). Meso scale discovery and luminex comparative analysis of calbindin D28K. J Biomed Biotechnol.

[12] Stefura WP, Graham C, Lotoski L, HayGlass KT (2019). Allergy Methods in Molecular Biology Chapter 7.

[13] Dabitao D, Margolick JB, Lopez J, Bream JH (2011). Multiplex measurement of proinflammatory cytokines in human serum: comparison of the Meso Scale Discovery electrochemiluminescence assay and the cytometric bead array. J Immunol Methods.

[14] Partridge MA, Purushothama S, Elango C, Lu Y. Emerging technologies and generic assays for the detection of anti-drug antibodies. J Immunol Res. 2016:1–6. 10.1155/2016/6262383. 10.1155/2016/6262383 PMC4983396 27556048

[15] He Y, Zhang M (1997). The positive and negative Symptom Scales (PANSS) and its application. J Clin Psychiatry.

[16] Zhang M (1984). The Brief Psychiatric Rating Scales (BPRS). Shanghai Psychiatry.

[17] Richard MD, Brahm NC (2012). Schizophrenia and the immune system: pathophysiology, prevention, and treatment. Am J Health-System Pharm.

[18] Benros ME, Mortensen PB (2019). Neuroinflammation and Schizophrenia Current Topics in Behavioral Neurosciences Chapter 93.

[19] Upthegrove R, Manzanares-Teson N, Barnes NM (2014). Cytokine function in medication-naive first episode psychosis: a systematic review and meta-analysis. Schizophr Res.

[20] Lesh TA (2018). Cytokine alterations in first-episode schizophrenia and bipolar disorder: relationships to brain structure and symptoms. J Neuroinflamm.

[21] Miller BJ, Buckley P, Seabolt W, Mellor A, Kirkpatrick B (2011). Meta-analysis of cytokine alterations in schizophrenia: clinical status and antipsychotic effects. Biol Psychiatry.

[22] Goldsmith DR, Rapaport MH, Miller BJ (2016). A meta-analysis of blood cytokine network alterations in psychiatric patients: comparisons between schizophrenia, bipolar disorder, and depression. Mol Psychiatry.

[23] Subbanna M (2018). Role of IL-6/RORC/IL-22 axis in driving Th17 pathway mediated immunopathogenesis of schizophrenia. Cytokine.

[24] Subbanna M (2019). Impact of antipsychotic medication on IL-6STAT3 signaling axis in peripheral blood mononuclear cells of drug‐naive schizophrenia patients. J Neuropsychiatry Clin Neurosci.

[25] Capuzzi E, Bartoli F, Crocamo C, Clerici M, Carrà G (2017). Acute variations of cytokine levels after antipsychotic treatment in drug-naïve subjects with a first-episode psychosis: a meta-analysis. Neurosci Biobehav Rev.

[26] Zhao ML, Si Q, Lee SC (2003). IL-16 expression in lymphocytes and microglia in HIV-1 encephalitis. Neuropathol Appl Neurobiol.

[27] Ahmad SF (2018). Elevated IL-16 expression is associated with development of immune dysfunction in children with autism. Psychopharmacology.

[28] Pawlowski T (2014). Depression and neuroticism in patients with chronic hepatitis C: correlation with peripheral blood mononuclear cells activation. J Clin Virol.

[29] Motta M, Imbesi R, Di Rosa M, Stivala F, Malaguarnera L (2007). Altered plasma cytokine levels in Alzheimer’s disease: correlation with the disease progression. Immunol Lett.

[30] Di Rosa M (2006). Chitotriosidase and inflammatory mediator levels in Alzheimer’s disease and cerebrovascular dementia. Eur J Neurosci.

[31] Duarte-Guterman P (2020). Inflammation in Alzheimer’s disease: do sex and APOE matter?. J Alzheimers Dis.

[32] Shinko Y (2020). Chemokine alterations in the postmortem brains of suicide completers. J Psychiatr Res.

[33] Dahan S (2018). The relationship between serum cytokine levels and degree of psychosis in patients with schizophrenia. Psychiatry Res.

[34] Xiu MH (2014). Decreased interleukin-10 serum levels in first-episode drug-naïve schizophrenia: relationship to psychopathology. Schizophr Res.

[35] Ding M (2014). Activation of Th17 cells in drug naïve, first episode schizophrenia. Prog Neuropsychopharmacol Biol Psychiatry.

[36] Goldsmith DR (2018). TNF-α and IL-6 are associated with the deficit syndrome and negative symptoms in patients with chronic schizophrenia. Schizophr Res.

[37] Garcia-Alvarez L (2016). Biomarcadores sanguíneos diferenciales de las dimensiones psicopatológicas de la esquizofrenia. Revista de Psiquiatría y Salud Mental.

[38] Li A (2020). A neuroimaging biomarker for striatal dysfunction in schizophrenia. Nat Med.

[39] Notter T (2018). Biomarkers in Psychiatry Current Topics in Behavioral Neurosciences Chapter 43.

[40] Yan J, Chen Y, Ju P, Gao J, Zhang L, Li J, Wang K, Zhang J, Li C, Xia Q, Zhu C, Zhang X (2022). Network association of biochemical and inflammatory abnormalities with psychiatric symptoms in first-episode schizophrenia patients. Front Psychiatry.

[41] Enache D, Nikkheslat N, Fathalla D, Morgan BP, Lewis S, Drake R (2021). Peripheral immune markers and antipsychotic non-response in psychosis. Schizophr Res.

[42] Pae C, Yoon C, Kim T, Kim J, Park S, Lee C (2005). Antipsychotic treatment may alter T-helper (TH) 2 arm cytokines. Int Immunopharmacol.

